# Prospective evaluation of two screening methods for molecular testing of metastatic melanoma: Diagnostic performance of BRAF V600E immunohistochemistry and of a *NRAS-BRAF* fully automated real-time PCR-based assay

**DOI:** 10.1371/journal.pone.0221123

**Published:** 2019-08-15

**Authors:** Audrey Vallée, Marie Denis-Musquer, Guillaume Herbreteau, Sandrine Théoleyre, Céline Bossard, Marc G. Denis

**Affiliations:** 1 Department of Biochemistry, Nantes University Hospital, Nantes, France; 2 Department of Pathology, Nantes University Hospital, Nantes, France; Ohio State University Wexner Medical Center, UNITED STATES

## Abstract

Screening for theranostic biomarkers is mandatory for the therapeutic management of cutaneous melanoma. *BRAF* and *NRAS* genes must be tested in routine clinical practice. The methods used to identify these alterations must be sensitive to detect mutant alleles in a background of wild type alleles, and specific to identify the correct mutation. They should not require too much material, since in some cases the available samples are small biopsies. Finally, they should also be quick enough to allow a rapid therapeutic management of patients. Sixty five consecutive formalin-fixed paraffin-embedded (FFPE) melanoma samples were prospectively tested for BRAF mutations with the VE1 (anti-BRAF V600E) antibody and for both *BRAF* and *NRAS* mutations with the Idylla NRAS-BRAF-EGFR S492R Mutation Assay cartridges. Results were compared to our routine laboratory practice, allele specific amplification and/or Sanger sequencing and discordant cases confirmed by digital PCR. Excluding discordant by-design-mutations, system failures and DNA quantity or quality failures, BRAF IHC demonstrated an overall concordance of 89% for BRAF V600E mutation detection, the Idylla system gave a concordance of 100% for *BRAF* mutation detection and of 92.1% for *NRAS* mutation detection when compared to our reference. When discrepancies were observed, all routine results were confirmed by digital PCR. Finally, BRAF IHC positive predictive value (PPV) was of 82% and negative predictive value (NPV) of 92%. The Idylla cartridges showed a PPV and NPV of both 100% for *BRAF* mutation detection and a PPV and NPV of 100% and 87% respectively, for *NRAS* mutation detection. In conclusion, BRAF V600E immunohistochemistry is efficient for detecting the V600E mutation, but negative cases should be further evaluated by molecular approaches for other *BRAF* mutations. Since 3 NRAS mutations have not been detected by the Idylla NRAS-BRAF-EGFR S492R Mutation Assay, these cartridges should not be used as a substitute for traditional molecular methods in the conventional patient therapeutic care process without the expertise needed to have a critical view of the produced results.

## Introduction

Screening for theranostic biomarkers is mandatory for the therapeutic management of many types of cancer such as lung cancer, colorectal cancer and melanoma. In cutaneous melanoma, mutations of the *BRAF* and *NRAS* oncogenes are the most common genetic alterations with mutation rates of ~40% and ~20%, respectively [1, 2]. Patients bearing a *BRAF* V600 mutation may benefit from BRAF and MEK inhibitors, but there is currently no approved targeted therapy for patients harboring *NRAS* mutation [3, 4]. Nevertheless, NRAS is a prognostic marker and a mechanism of acquired resistance to BRAF inhibitors [5]. Therefore both *BRAF* and *NRAS* genes should be tested in routine clinical practice, along with *KIT* for mucosal and acral melanoma [6–8].

The methods used to identify these alterations must be sensitive to detect mutant alleles in a background of wild type alleles, and specific to identify the correct mutation. They should not require too much material, since in some cases the available samples are small biopsies, which are fixed in formalin and embedded in paraffin (FFPE). Finally, they should also be quick enough to allow a rapid therapeutic management of patients.

For *BRAF* V600 testing, several methods have been developed: protein-based analysis and DNA-based analysis [9]. Protein-based analyses are mostly represented by IHC, using the monoclonal antibody VE1, validated to recognize the mutant BRAF V600E protein [10]. DNA-based analyses include real-time PCR based assays [11, 12], pyrosequencing [13] and Next Generation Sequencing [14]. Real-time PCR assays are usually multiplexed amplifications designed to scan the most relevant and frequent therapeutic mutations while new generations of sequencing cover a large panel of mutations even beyond the therapeutic targets.

The turnaround-time of these methods is also of importance since treatment decisions are based on the results obtained. Therefore, rapid techniques detecting the most frequent therapeutic biomarker alterations are attractive. BRAF IHC has been proposed as a screening tool for *BRAF* V600E mutation [13, 15, 16]. A fully automated real-time PCR platform (Idylla system) has been designed to perform molecular analysis in ~2 hours. The Idylla *BRAF* Mutation Assay has been shown to be efficient for testing FFPE melanoma samples [17–19]. Recently, new cartridges have been designed to allow the simultaneous detection of the most relevant *BRAF* and *NRAS* clinical alterations in a single assay, which is of interest for colorectal tumors and melanoma. Very few data are available, and most of these comparative studies have been conducted on selected FFPE samples [20–22].

We thus conducted a prospective study comparing the performance of BRAF Immunochemistry and Idylla NRAS-BRAF-EGFR S492R Mutation Assay (RUO) to our standard reference methods for *BRAF* and *NRAS* mutations detection. We report here the results obtained on 65 consecutive unselected melanoma FFPE samples tested using these approaches.

## Materials and methods

### Patient samples

Sixty five consecutive FFPE tumor samples from 60 patients with metastatic melanoma were collected prospectively. Tumor content was assessed on hematoxylin- & eosin-stained sections. Serial 10-μm tumor sections were prepared for molecular analysis. At least one section was used for molecular analysis according to our routine procedures. An additional section was used for testing on the Idylla platform. When possible, BRAF immunohistochemistry was performed using a remaining section. Tumor area was measured using the open source ImageJ software (NIH, Bethesda, Maryland). All assays were processed independently and blinded to mutation status determined by the other methods. Results were pooled for comparison at the end of the study.

The Ethics Committee (Comité de Protection des Personnes/CPP) considered that neither patient consent nor CPP approval was required for this non-interventional study.

### DNA extraction

For routine *BRAF*/*NRAS* testing, DNA was purified following paraffin removal and macrodissection using the Maxwell RSC DNA FFPE kit on a Maxwell RSC system (Promega, Charbonnières-les-Bains, France). DNA concentration was quantified by spectrophotometry (NanoDrop ND-100 instrument, Thermo Fisher Scientific, Waltham, MA) and diluted to a final concentration of 5 ng/μl.

### Detection of BRAF V600 mutations with allele-specific amplification

The most frequent *BRAF* mutations were detected by allele-specific amplification (ASA) as previously described [12] with minor modifications. We designed one common reverse primer (BRAF_AS; ATGGATCCAGACAACTGTTCAAAC) and two forward primers with a unique variation in their 3’ nucleotide such that each was specific for the wild type (V600; AGGTGATTTTGGTCTAGCTACAGT) or the mutated variant (600E; AGGTGATTTTGGTCTAGCTACAGA). Hence, 2 different PCR mixes were prepared: one for wild type allele detection and the second for mutant allele detection. Each mix contained 10 μl of LC480 Sybr green 2x master mix (Roche Diagnostics, Meylan, France), 0.5 μl of each primer (10 μM each), and 9 μl of the template (45 ng genomic DNA). Amplification conditions were optimized for the RotorGeneQ instrument (QIAGEN, Courtaboeuf, Ozyme, Saint Quentin en Yvelines, France) as follows: denaturation for 10 min at 95°C; amplification for 45 cycles, with denaturation for 10 s at 95°C, annealing for 15 s at 65°C, and extension for 20 s at 72°C.

The difference between the Ct values (ΔCt) of mutant and wild type allele amplifications was calculated. The lower the amount of mutated DNA in the sample, the higher the ΔCt value. This result was compared to a threshold which discriminates specific mutant amplification of PCR background. This ΔCt threshold was set at 7.

This assay can detect (but not distinguish) the V600E (c.1799T>A), the V600K (c.1798_1799GT>AA) and the V600D (c.1799_1800TG>AT) mutations, but not the V600R (c.1798_1799GT>AG) mutation. Therefore each sample was further analyzed by conventional Sanger DNA sequencing for nucleotide characterization or detection of *BRAF* mutations outside the codon 600 hotspot.

### Detection of *BRAF* exon 15 and *NRAS* exon 2 and 3 mutations by Sanger sequencing

PCR amplifications were performed using the following primers: BRAF 15F (5’-TCATAATGCTTGCTCTGATAGGA-3’) and BRAF 15R (GGCCAAAAATTTAATCAGTGGA) for BRAF exon 15, NRAS 2F (5’-CCCCCAGGATTCTTACAGAA-3’) and NRAS 2R (5’-ATACACAGAGGAAGCCTTCG-3’) for NRAS exon 2; NRAS 3F (5’-CCCCTTACCCTCCACACC-3’) and NRAS 3R (5-TGGCAAATACACAGAGGAAGC -3) for NRAS exon 3. All primers harbored universal M13 tags at their end. Cycling conditions were as follows: denaturation for 10 min at 95°C; amplification for 40 cycles, with denaturation for 20 s at 95°C, annealing for 30 s at 60°C, and extension for 10 s at 72°C. Sanger sequencing was performed using the universal M13 forward and reverse primers and Big Dye Terminator Chemistry v1 on an ABI3130XL Instrument. Sequences were analyzed using the Seqscape Software (Applied Biosystems, Thermo Fisher Scientific, Waltham, MA).

### BRAF immunohistochemistry

Immunohistochemistry was performed on 5-μm formalin-fixed paraffin-embedded (FFPE) sections from the same tissue block used for molecular testing. Slides were stained with anti-BRAF V600E mutant-specific antibody (clone VE1, dilution 1:200, pH9, Eurobio) *[10]*. The immunological reaction was visualized with the Envision detection system (Dako) and AEC (3-amino-9 ethylcarbazole) as the chromogen, allowing the detection of VE1+ tumor cells even if these cells contained cytoplasmic melanin pigment. The sections were counterstained with Mayer’s hematoxylin. As a negative control, primary antibody was omitted. A BRAF V600E mutated melanoma served as positive external control on each immunostained slide. All Immunostainings were analyzed by two pathologists (MDM and CB) blinded to genetic data, and scored as follows: positive (with the percentage of VE1+ tumor cells) when viable tumor cells harbored cytoplasmic staining, negative when no staining or only scarce VE1+ tumor cells or only VE1+ macrophages.

### Detection of *BRAF* exon 15 and *NRAS* exons 2, 3 and 4 mutations with the Idylla molecular diagnostic system

The Idylla system (Biocartis, Mechelen, Belgium) is a fully automated real-time PCR-based system for molecular diagnostics. Single-use cartridges contain all the necessary reagents to perform sample lysis, DNA extraction and real-time PCR amplification. The Idylla console software analyses the fluorescence signals and reports the presence or absence of a mutation. The Idylla NRAS-BRAF-EGFR S492R Mutation Assay cartridges are designed to detect 25 different mutations: five mutations in codons 600 of the *BRAF* gene, 8 mutations in codons 12 and 13; 6 in codons 59 and 61; 4 in codons 117 and 146 of the *NRAS* gene and 2 mutations in codon 492 of the *EGFR* gene.

All tumor areas were macrodissected to be comparable to the laboratory’s standardized process. Samples which did not meet the recommendation (area) were not excluded. FFPE material was placed between two filter papers and transferred to the cartridge as per the manufacturer’s procedure. Finally, the cartridge was loaded onto the Idylla system for processing. All results were exported from the Biocartis console.

### Mutation detection by digital PCR

Discordant samples were analyzed using a chip-based digital PCR platform, the Quant Studio 3D (Life Technologies, Thermo Fisher Scientific, Waltham, MA). The chip consists of 20.000 PCR wells in which DNA and PCR reagents are distributed. Taqman probe technology allows the detection of wild-type or a mutated or both DNA copies in each well. Fluorescence signal processing associated with the Poisson distribution allows absolute quantification of mutated and wild type copies. Mutation frequencies are determined by the ratio of mutated signals to wild type signals. This technique is considered more sensitive and accurate than conventional PCR methods, especially for the detection of low-frequency variants [23, 24].

The dPCR mastermix and Taqman assays were purchased from Thermo-Fisher Scientific. Twenty-five nanograms of FFPE DNA were added to the digital PCR mix before automatic distribution to a chip. After PCR amplification, raw data collected from the fluorescent reader were analyzed with a Visual Basic Application on Excel developed by our laboratory (unpublished data).

## Results

### Prospective study cohort

Sixty five consecutive FFPE tumor samples, collected from 60 patients with metastatic melanoma, were collected prospectively, representing the diversity of our laboratory’s routine recruitment (Table 1). Patient median age was 69 years (range 22–91). Most samples were surgical resections (n = 42, 65%). Both primary tumors (n = 32, 49%) and metastases (n = 32, 49%) were tested. In most cases, the tumor cell content of FFPE tissue sections was greater than 50% (n = 52, 80%), and the tumor area larger than 25 mm^2^ (n = 41; 63%)(S1 Table), the minimum tumor area required for an Idylla test. The 23 samples (35%) that did not meet this criterion were still analyzed by all three techniques.

**Table 1:** Characteristics of the 60 patients with metastatic melanoma and the 65 corresponding tumor samples tested.

	N (%)
Gender	
Male	38 (63)
Female	22 (37)
Age	
≤ 69	32 (53)
>69	28 (47)
**Total patients**	60 (100)
Tumor tissue origin	
Primary tumor	32 (49)
Metastatis	32 (49)
NA/ND	1 (2)
Sample Nature	
Biopsy	22 (34)
Surgical sample	42 (65)
NA/ND	1 (1)
% of tumor cells	
>50%	52 (80)
25–50%	9 (14)
10–25%	4 (6)
<10%	0 (0)
Tumor area	
<25 mm^2^	34 (52)
>25 mm^2^	30 (46)
NA	1 (2)
Total area available for testing*	
<25 mm^2^	23 (35)
>25 mm^2^	41 (63)
NA	1 (1,5)
**Total samples**	65 (100)

NA, information not available; ND, not determined

* Tumor area x number of tissue sections

### Performance of BRAF testing methods

The results obtained with the 3 different techniques are presented in Table 2. All 65 samples were processed for *BRAF* testing with the techniques used in routine practice in our hospital: allele-specific amplification and Sanger sequencing. Complete results were obtained for 63 samples. The two remaining samples could only be assessed by ASA as DNA sequencing was not contributive. These sequencing failures were mostly due to poor DNA quality, complicating amplification of the 224-pb fragment for *BRAF* sequencing, whereas the ASA fragment was only 75-pb long. Using this approach, *BRAF* mutations were detected in 23 out of the 65 samples (35%), which is in line with the frequency of *BRAF* mutations in metastatic melanoma [1, 2].

**Table 2:** Comparison of BRAF and NRAS results by allele specific amplification, Sanger sequencing, IHC and Idylla testing.

		**BRAF IHC**				**BRAF Idylla**				
		N	P	NT	**All**	No mutation	BRAF V600	Insufficient material	Cartridge failure	**All**
BRAF ASA +Sanger sequencing	WT	38	0	4	42	41		1		42
	BRAF V600X*	0	1	0	1	1				1
	BRAF V600E	3	13	1	17		17			17
	BRAF V600K	5	0	0	5		4		1	5
	**All**	46	14	5	65	42	21	1	1	65
* *		**NRAS Idylla**						** **	** **	
		WT	NRAS Q61X	NRAS G13X	Insuff. material		Cartridge failure	**All**			
NRAS Sanger sequencing	WT	20					20			
	NT (BRAF mutated)	22				1	23			
	NRAS Q61R	2	7				9			
	NRAS Q61K		5				5			
	NRAS Q61L	1	2				3			
	NRAS Q61_E62delinsHK	1					1			
	NRAS G13R			1			1			
	NRAS G60E	1					1			
	Not Contributive	1			1		2			
	**All**	48	14	1	1	1	65			

N, Negative; P, Positive; NT, Not Tested

* Nucleotide characterisation by Sanger sequencing failed

These 65 samples were also tested using the Idylla NRAS-BRAF-EGFR S492R Mutation Assay cartridges. The test was not contributive in 2 cases (3,1%): one test failed due to insufficient material and one due to a software error. *BRAF* mutations were detected in 21 out of the 63 contributive samples (33%).

Finally 60 of the 65 samples were available for BRAF V600E assessment by IHC. A *BRAF* mutation was detected in 17 of the 60 tested samples (28%). As expected BRAF IHC did not detect any of the *BRAF* c.1798_1799delinsAA (V600K) mutations.

**Fig 1:**
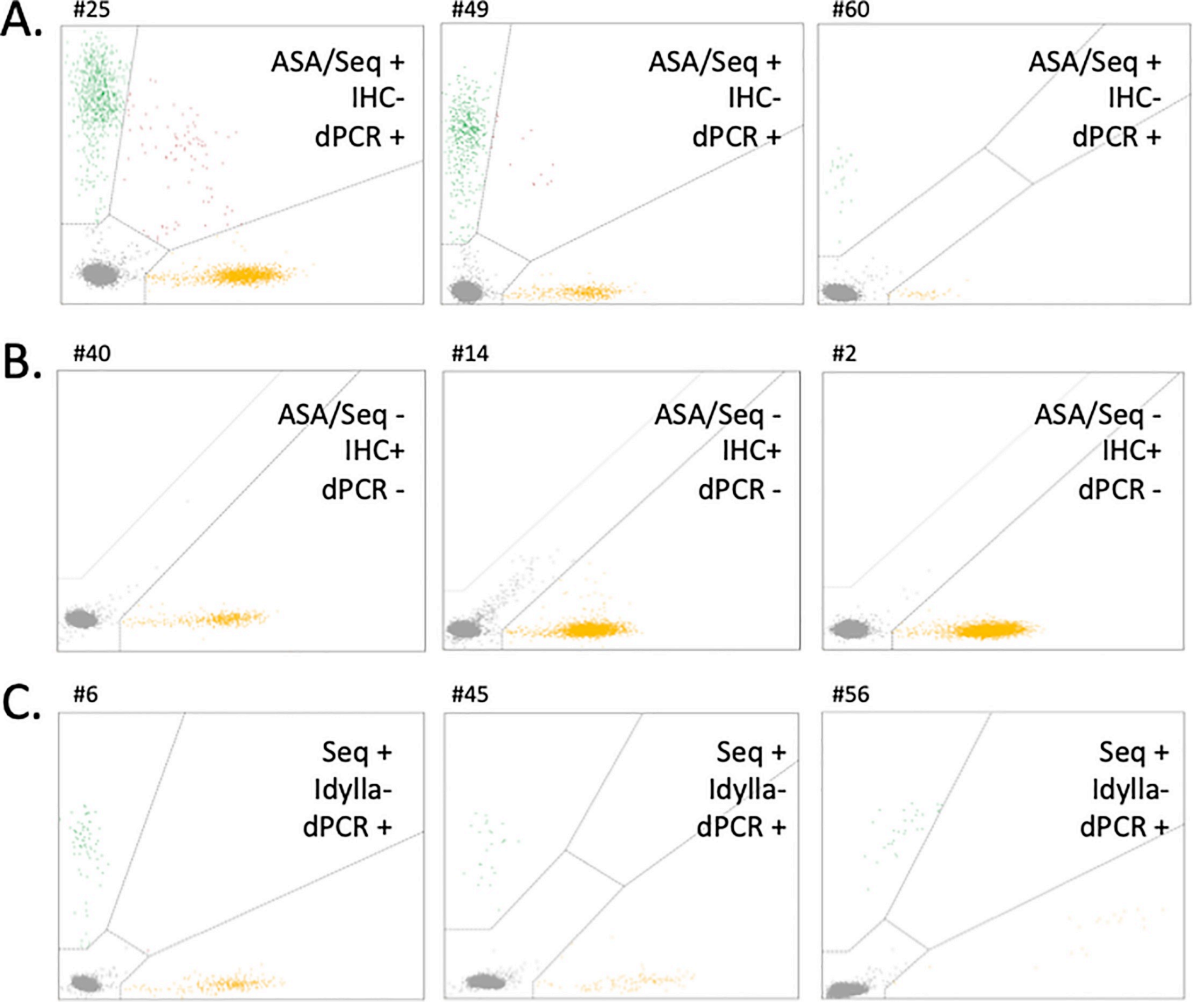
Discordance analyses by digital PCR. A. Control of samples found positive for *BRAF* mutation by ASA/Sequencing but negative by IHC. B. Control of samples found negative for *BRAF* mutation by ASA/sequencing and positive by IHC (wrong chromogen used). C. Control of samples found positive for *NRAS* mutation by sequencing but negative by Idylla. Yellow dots correspond to wild type DNA copies (*BRAF*, panels A and B; *NRAS*, panel C). Green dots correspond to mutated DNA copies (*BRAF* V600E, panels A and B; *NRAS* Q61R, panel C). Grey dots correspond to empty wells.

**Fig 2:**
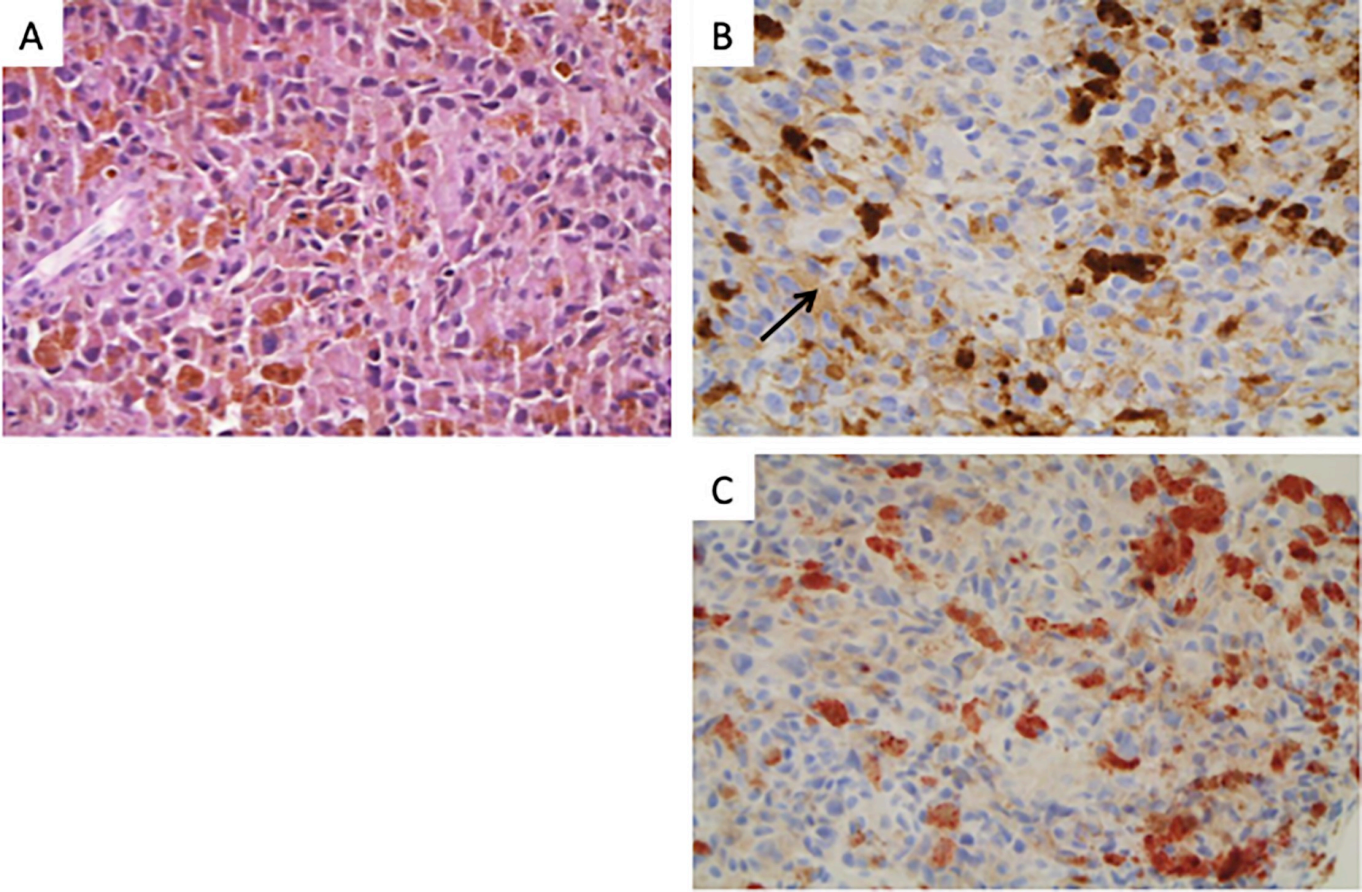
A representative BRAF IHC/In-house testing discordant case. Hematoxylin & eosin stained section (A) Some tumor cells admixed with numerous melanophages (arrow) harbor a weak/moderate BRAF immunostaining with DAB (B) which disappears with AEC (C).

The discrepancies observed were further investigated by digital PCR. This method allows both absolute and relative quantification of low variant allele frequency (VAF), ensuring equal if not better performance than ASA or Idylla testing. The results are presented in Fig 1. One sample (#23) could not be confirmed as the DNA was too degraded; otherwise, all in-house assay results were confirmed by digital PCR. Finally we observed 3 false *BRAF* negative results (Fig 1A) and 3 false *BRAF* positive results with IHC (Fig 1B). For these 3 latter cases, IHC was repeated and turned out to be negative on a second read. DAB (3,3’-Diaminobenzidine) instead of AEC had been used as chromogen on the first tissue sections, generating false positive staining (Fig 2). Therefore, these 3 cases were subsequently considered as true negative results (Table 2). The details of all confirmed discordant *BRAF* genotypes are presented on Table 3.

**Table 3:** Discordant genotyping results.

**BRAF discordances**							
		**Results**					
Sample number	Tumor area (mm^2^)	**In-house assay**	**Idylla**	**IHC**	**Digital PCR (VAF%)**		**Conclusion**
25	9	BRAF V600E	BRAF V600	BRAF Neg	BRAF V600E (30%)		IHC false negative result
49	50	BRAF V600E	BRAF V600	BRAF Neg	BRAF V600E (48%)		IHC false negative result
60	35	BRAF V600E	BRAF V600	BRAF Neg	BRAF V600E (32%)		IHC false negative result
23	190	BRAF V600X*	WT	BRAF Pos	DNA degraded. Digital PCR failure		Not conclusive
**NRAS discordances**							
** **		**Results**					
Sample number	Tumor area (mm^2^)	**In-house assay**	**Idylla**	**Digital PCR (VAF%)**			**Conclusion**
6	30	NRAS Q61R	NRAS WT	NRAS Q61R (17%)			Idylla false negative result
45	21	NRAS Q61R	NRAS WT	NRAS Q61R (15%)			Idylla false negative result
56	8	NRAS Q61L	NRAS WT	NRAS Q61L (58%)			Idylla false negative result

* Nucleotide characterisation by Sanger sequencing failed

For performance analysis, 5 discordant-by-design mutations (BRAF V600K) were excluded from BRAF IHC evaluation, and 2 from BRAF/NRAS Idylla test performance analysis. The cartridge failure, the sample with insufficient material and the result that could not be confirmed by digital PCR were also excluded. True and false positive or negative results are reported in Table 4. We conclude to a positive predictive value (PPV) of 100% and a negative predictive value (NPV) of 93% for BRAF V600E detection with IHC, a PPV and NPV of both 100% for *BRAF* mutation detection with Idylla.

**Table 4:** Performance of BRAF and NRAS testing by IHC and Idylla compared to in-house assay.

	BRAF IHC	BRAF Idylla	NRAS Idylla
True positive	14	21	15
False positive	0	0	0
True negative	38	41	20
False negative	3	0	3
Total	55	62	38
Positive Predictive Value	100%	100%	100%
Negative Predictive Value	93%	100%	87%

### Performance of the Idylla system for *NRAS* testing

*BRAF* and *NRAS* mutations are mutually exclusive in treatment naïve patients [2, 25]. Therefore, in our routine practice, we only perform *NRAS* sequencing for *BRAF* wt samples. Only the 42 *BRAF* wild type samples of this study cohort were sequenced for *NRAS* exon 2 and 3 mutations. Contributive results were obtained for 41 samples. The sample which failed for *NRAS* analysis also failed for *BRAF* sequencing. Considering the Idylla system, with *BRAF* and *NRAS* mutation detection being carried out on the same cartridge, as mentioned above, all 65 samples underwent a test but one cartridge failed to run and one test failed due to insufficient material (2/65; 3.1).

*NRAS* mutations were found in 20 out of the 41 valid samples (48.7%) with Sanger sequencing and 15 out of the 63 (23.8%) with Idylla or in 15 out of the 41 (36.6%) if we focus on samples that had been sequenced (Table 2). As expected, no *NRAS* mutations were found with Idylla on samples bearing a *BRAF* mutation. Three common alterations (2 Q61R mutations and 1 Q61L mutation) were found by sequencing but not by the Idylla system. Sanger sequencing also highlighted two mutations not covered by Idylla PCR probes: one complex mutation (Q61_E62delinsHK) and one rare variant of unknown clinical significance (G60E). Conversely, the NRAS-BRAF_EGFR S492R cartridge was initially designed for colorectal cancers and also screens *NRAS* exon 4. No alteration of this exon was detected with the cartridges.

Taking into account only the mutations tested by all approaches, the overall agreement between Sanger sequencing and Idylla NRAS-BRAF-EGFR S492R Mutation Assay was 92.1% (35/38).

Considering *NRAS* results, 3 discordances were observed between Sanger sequencing and Idylla (Table 3). These discrepancies were also investigated using digital PCR. Results are detailed in Table 3 and digital PCR data are presented in Fig 1. The DNA sequencing data were all confirmed by digital PCR. Therefore, we conclude that 3 false *NRAS* negative results were obtained with the Idylla cartridge (Fig 1C).

In summary, true and false positive and negative results are reported in Table 4. We conclude to a PPV and NPV of 100% and 87% respectively, for *NRAS* mutation detection using the Biocartis cartridges.

## Discussion

For our routine clinical practice, we have developed a workflow for molecular analysis of tumor samples from metastatic melanoma [26–28]. Using these tests, over the past 5 years, we have tested 1920 melanoma samples. Our laboratory is accredited in accordance with the International Standard ISO15189. The turnaround time (TAT) is usually between 3 and 7 days since the tests are performed once a week. Recently, rapid screening methods have emerged. These are designed to be used as soon as a sample is available, thus reducing the TAT. In this study we compared the performance of two of these rapid methods for the detection of mutations in melanoma samples.

BRAF immunohistochemistry is based on the use of the VE1 antibody, which only detects the BRAF V600E mutation [10]. BRAF IHC is presented as a pre-screening tool, allowing the rapid introduction of a treatment based on BRAF inhibitors [13, 29]. This is only feasible if the PPV is of 100%. In our prospective study, IHC yielded a final PPV of 100% and a NPV of 93% when calculated with V600E mutated samples only. Three tests were confirmed as false negative. This has already been described [16, 30]. In a routine workflow, negative samples would be further investigated using DNA-based techniques for other *BRAF* V600 mutations, which have also been shown to drive clinical benefit from BRAF inhibitors. In the initial analysis, 3 tests were false positive. This was linked to a technical error involving use of the wrong chromogen, and for this reason they were then considered as true negative. However it is important to point out that the interpretation based on IHC can be difficult, especially when the number of tumor cells is very low or when heterogeneous staining is observed [13, 15]. When focusing on these particular stains, the PPV of BRAF IHC dropped to 70% in the study performed by Tetzlaff et al. [15]. Hence, the vast majority of these studies recommend addressing equivocal or low sample staining for DNA-based molecular testing [13, 15, 29]. Furthermore, since *NRAS* testing is also of importance, procedures allowing the simultaneous analysis of both *BRAF* and *NRAS* are attractive.

In the current study, we also evaluated the Idylla platform. This is a fully automated real-time PCR-based system, capable of detecting most clinically relevant mutations. We tested the Idylla NRAS-BRAF-EGFR S492R Mutation Assay cartridges, originally designed for colorectal cancer, but also of interest for melanoma as it enables, in a single 2-hour run without any sample pre-treatment, the qualitative detection of 5 mutations in codon 600 of the *BRAF* gene, 18 mutations in codons 12, 13, 59, 61, 117 and 146 of the *NRAS* gene and 2 mutations in codon 492 of the *EGFR* gene. After exclusion of the mutations not tested by all techniques and various technical failures, the Idylla NRAS-BRAF-EGFR S492R Mutation Assay displayed a PPV and NPV of 100% for *BRAF* mutation assessment. The assay was less accurate for the detection of *NRAS* mutations since the final PPV was 100%, but the NPV was 87%. This lower NPV result was due to 3 false negative results. These samples were confirmed by dPCR to harbor two *NRAS* Q61R mutations and one *NRAS* Q61L mutation at allele frequencies of 15%, 17% and 58%, which is above the expected sensitivity of the Idylla NRAS-BRAF-EGFR S492R Mutation Assay (ranging between 1 and 5% depending on the mutation). The tumor cell content of these samples was comprised between 25% and 50% for 2 samples, and above 50% for the third one. Tumor areas loaded into the cartridges were 32, 42 and 60 mm^2^, which is in the range of what is recommended by the manufacturer (25–300 mm^2^ for 10-μm sections).

Three previous studies reported their results using the NRAS-BRAF-EGFR or NRAS-BRAF Mutation Assay in colorectal cancer [21, 22, 31] and one in metastatic melanoma [20]. These evaluations were conducted on archival FFPE samples selected according to size and mutational status. Three found a complete agreement of *BRAF* results with NGS results [20, 21] or MassARRAY [22] and the only multicenter and multi-molecular method evaluation found 2 false positives (2 of 322 negative samples; n = 410; PPV 98%; NPV 99%) [31]. Considering *NRAS* results, the same study reported 1 false positive (1/319) and 4 false negative results (4/94), including two samples that were finally excluded from the performance analysis as the allele frequency obtained with the confirmation method was under the LOD of the Idylla system. In other monocenter studies, when compared to NGS and Mass Array [20–22], the Idylla system demonstrated a greater or similar sensitivity. However, Barel et al. noticed one difference in mutation genotyping since NGS highlighted an *NRAS* G13C mutation confirmed by SNaPshot whereas the Idylla cartridges, which do not search the G13C alteration, reported a *NRAS* G12A.

Though Idylla *BRAF* analysis confirmed through these results and our own study to be sensitive and quite specific, we reported an inferior sensitivity of *NRAS* mutation detection, also observed in the Prieto-Potin team results, though to a lesser extent. As hypothesized by Barel et al. [20], *NRAS* alterations are more numerous than *BRAF* alterations and primer multiplexing is probably more challenging for a fully automated system requiring the use of stringent internal cut-off which could lead to a decreased sensitivity. The reason for our greater lack of sensitivity is probably due to the prospective nature of our cohort, and to the absence of any selection bias. All the samples collected were tested, independently of their size. By contrast for instance, to select 235 archival FFPE samples, Johnston et al. excluded 11 samples for insufficient material and 8 because the tumor area did not meet the Idylla criteria [22].

The tumor area required to use the cartridges is 50–600 mm^2^ of 5-μm FFPE sections. If we had strictly applied these requirements, 23 samples out of 65 (35.4%) would have been excluded from our cohort. In routine practice, additional tissue sections (when possible) or alternative techniques would have been necessary. It should be noted that concordant results have been obtained on samples with lower amounts of material than the requirements, but in a context of a certified laboratory, the use of a reagent outside the manufacturer’s protocol should be strongly validated and documented.

Besides the tumor area, a second major limitation is the impossibility to collect DNA from the cartridge after test completion. In our study, 11 out the 65 patients required a *KIT* gene analysis. Thereby more sample sections would have been necessary for further molecular analysis consuming more material on sometimes small samples. Moreover, in the event of assay failure, one or more new FFPE sections would be necessary. Jonhston et al. had to repeat up to 4 cartridges (4/235; 1.74%) after instrument errors [21, 22].

Finally, cost issues are an important factor. Several studies have demonstrated that the Idylla platform has the shortest hands-on time compared to conventional molecular methods [32]. The cost of an Idylla NRAS-BRAF Mutation test was about €216 in France in 2017 [33], with almost no labor cost. We evaluated the overall cost of our routine tests at €36 (reagents €21; labor 15€). Bisschop et al. [32] estimated the cost of reagents for different techniques (list prices not including salary and equipment): in-house methods HRM/Sanger (€175), Next Generation sequencing (€275), ddPCR (€45) and BRAF IHC (€122). IHC only identifies the BRAF V600E mutation, whereas dPCR (using multiplex assays or as separate reactions with different probes) and Idylla can be used to detect the most common BRAF mutations. NGS provides a larger molecular profile of targetable biomarkers. Hence the ratio cost/information provided should be analyzed carefully.

With a very easy laboratory implementation and a fast turnaround time, the Idylla system is an attractive tool for fast therapeutic marker detection, especially for rapidly progressive patients. Nevertheless, the Idylla NRAS-BRAF-EGFR S492R Mutation Assay has demonstrated a limited sensitivity for the detection of some NRAS mutations and the material requirement could be problematic for small tissue fragments, compromising the opportunity of additional molecular tests. We concluded that the Idylla NRAS-BRAF-EGFR S492R Mutation Assay should not be a substitute for traditional molecular methods in a conventional therapeutic patient care process without the expertise needed to have a critical view of the produced results.

## Supporting information

S1 Table Characteristics of the 65 samples tested with the Idylla NRAS-BRAF-EGFR S492R Mutation Assay cartridges. Tumor areas were measured using the open source ImageJ software.
(XLSM)
